# Evaluation of olfactory function in patients undergoing endoscopic skull base surgery with nasoseptal flap

**DOI:** 10.1016/j.bjorl.2020.03.006

**Published:** 2020-04-27

**Authors:** Ana Carolina Mayor de Carvalho, Ricardo Landini Lutaif Dolci, Jeniffer Cristina Kozechen Rickli, Daniela Akemi Tateno, Davi Sousa Garcia, Williams Escalante Encinas, Américo Rubens Leite dos Santos, Paulo Roberto Lazarini

**Affiliations:** a Santa Casa de Misericórdia de São Paulo, Departamento de Otorrinolaringologia, São Paulo, SP, Brazil; b Faculdade de Ciências Médicas da Santa Casa de São Paulo, São Paulo, SP, Brazil; c Santa Casa de Misericórdia de São Paulo, Departamento de Cirurgia, Disciplina de Neurocirurgia, São Paulo, SP, Brazil; d Universidade de Fortaleza (Unifor), Faculdade de Medicina, Fortaleza, CE, Brazil

**Keywords:** Nasal surgical procedure, Skull base, Smell, Surgical flap, Olfaction disorders

## Abstract

**Introduction:**

Endoscopic transnasal access to the skull base, both for treatment and reconstruction, can cause olfactory morbidity. Knowing the main consequences of this intervention is essential to have objective criteria for decision-making regarding the appropriate surgical technique.

**Objectives:**

The aim of this study is to determine the impact on olfactory function of the endonasal endoscopic access to the skull base with the creation of the nasoseptal flap.

**Methods:**

A prospective research was carried out in which 22 patients who underwent endoscopic transnasal surgery at the skull base, with the creation of a nasoseptal flap. The Connecticut Chemosensory Clinical Research Center test was applied before and at the 1^st^, 3^rd^ and 6^th^ postoperative months.

**Results:**

The results showed that only in the first month of follow-up the mean patient classification was statistically worse than at the other evaluation moments (*p* < 0.05), but there was no mean difference in the Connecticut score classification between the other moments (*p* > 0.05); that is, patients showed worsening in the 1^st^ month and returned to the preoperative mean after the 3^rd^ month of follow-up.

**Conclusion:**

The present study showed that the postoperative decrease in olfaction is transient, since the patient's sense of smell returns to pre-surgical values in the 3^rd^ postoperative month.

## Introduction

Endoscopic transnasal surgeries at the skull base, both in the stage of tumor excision and in the skull base reconstruction, can compromise natural physiological processes, consequently increasing postoperative sinonasal morbidity, with a negative impact on patient quality of life.^1^, ^2^

The olfactory neuroepithelium is located in the upper recess of the nasal cavities, extending through the cribriform plate, upper third of the nasal septum and through regions of the upper and middle turbinates.^3^ To perform the endoscopic transnasal access to the skull base, structures that contain this epithelium are removed, increasing the risk of olfactory dysfunction, which is ultimately among the main consequences of this surgery.^4^

Additionally, the Nasoseptal Flap (NSF), used in the reconstruction of the skull base defect and prevention of cerebrospinal fluid (CSF) fistula, can aggravate the reduction in the olfaction, since, for its creation, the olfactory neuroepithelium or regions close to it must be manipulated.^5^

The transnasal endoscopic route at the skull base has shown to be a favorable access route. However, it can have a significant impact on olfaction. Knowing the main consequences of this intervention, which is still the subject of few studies, is essential to have objective elements for decision-making regarding the appropriate surgical technique, aiming to minimize postoperative morbidity.

## Objectives

The aim of this study is to determine the impact of the endonasal endoscopic access to the skull base on olfactory function, using the Connecticut Chemosensory Clinical Research Center (CCCRC) smell test indexes.

## Methods

A prospective study was carried out between May 2015 and January 2017 including patients with skull base tumors, candidates for endoscopic transnasal surgery for access to the sella turcica or expanded endonasal access to the base.

The inclusion criteria were: endoscopic access to the skull base via the transnasal route, creation of the nasoseptal flap, partial removal of the middle turbinate, anterior and posterior ethmoidectomy ipsilateral to the middle turbinectomy, creation of the reverse flap and posterior septectomy from the posterior chondrotomy. As for the exclusion criteria, they included patients unable to correctly answer the applied CCCRC smell test, patients who had anosmia prior to the surgery or patients submitted to transcribriform access.

The pathologies identified in these patients were Adrenocorticotropic Hormone (ACTH)- or Growth Hormone (GH)-Producing Pituitary Adenomas, non-hormone-producing pituitary adenomas, craniopharyngioma, tuberculum sellae meningioma or lateral recess meningoencephalocele. The surgery was performed by the same team of otorhinolaryngologists and neurosurgeons of the same institution.

Olfaction evaluation was performed in all patients using threshold and smell identification tests based on the CCCRC test. The tests were performed in the 24 h prior to surgery and repeated 1, 3 and 6 months after the surgical procedure. In the meantime, patients received regular follow-up by the Otorhinolaryngology team to clean the nasal cavities, remove crusts and resect synechiae.

To carry out the statistical analysis of the study, first the two groups (transsellar access and expanded access) were compared in relation to the behavior during the study, to elucidate whether they had the same or different behaviors over time. Then, after evaluating this topic, which showed that both groups behaved in the same way, we chose to join them again into a single group and carry out the evaluation of olfaction variation over time.

The study was submitted to and approved by the institution's Research Ethics Committee (Approval Protocol number in the Ethics Committee: 31589414.5.0000.5479).

### Surgical technique

The present study is based on the techniques of endonasal endoscopic access to the sella turcica and expanded endonasal endoscopic access to the skull base, both with the creation of a nasoseptal flap.

#### Creation of the nasoseptal flap

The nasoseptal flap, described in 2006 by Hadad et al., consists of using a pedunculated mucosal flap from the nasal septum supplied by the posterior nasoseptal artery (posterior branch of the sphenopalatine artery). Removal of the middle turbinate, ipsilateral anterior and posterior ethmoidectomy for skull base access facilitates the visualization of the vascular pedicle and elevation of the flap, as well as increasing the space for four-hand work.^6^

The flap is created according to the size and shape of the anticipated defect. Two parallel incisions are made in the nasal septum mucosa through the chosen nasal fossa. The first (lower) incision should be made parallel to the nasal fossa floor, from posterior to anterior between the septum and the floor. If the defect in the skull base is a wide one, it is possible to extend this incision to the lower turbinate region. The second (upper) incision should be made respecting the distance of 1.5–2 cm from the upper region of the nasal septum to the middle turbinate region, for maximum preservation of the olfactory neuroepithelium. The two horizontal incisions must be joined anteriorly by a vertical incision and extended posteriorly toward the sphenoid rostrum. The lower incision continues with a vertical incision bordering the lower edge of the choana and extending laterally. The upper incision follows immediately below the sphenoid sinus ostium toward the lateral nasal wall. Thus, a posterior pedicle is created, involving the posterior nasoseptal artery.

The elevation of the mucoperichondrial and mucoperiosteal flap is initiated anteriorly to the posterior region, leaving a postero-lateral pedicle. Once elevated from the septum, the flap can be positioned in the nasopharynx until the surgical phase of tumor access and removal has been completed. In these endoscopic accesses to the skull base, the posterior portion of the nasal septum is removed after the flap is created.

#### Reverse flap

After the creation of the nasoseptal flap, the reverse flap is created, which aims to reduce the formation of crusts in the bare septal region on the side where the nasoseptal flap was created.

With that, two posterior incisions (upper and lower) are made, starting right after the remainder of the nasal septum, which follow toward the nasal cavity. Incision communication occurs later, close to the sphenoid rostrum region, through a vertical incision. Then, this nasal mucosal flap is pulled in the anterior direction, covering the entire bare septal region, which provides for earlier epithelialization. Finally, this mucosa is sutured with 4.0 absorbable suture and a nasal splint is placed, which is fixed over the bilateral reverse flap with 3.0 non-absorbable suture thread and removed 14 days after the surgery.^7^

#### Olfactory area

The olfactory neuroepithelium is distributed in the region of the cribriform lamina, middle, upper and supreme turbinate (when present) and the upper portion of the nasal septum (olfactory strip). In this region, the anterior limit extends to the anterior insertion of the middle turbinate. Therefore, the upper incision made in the nasal septum, after passing the middle turbinate, does not need to preserve 1.5–2 cm of the septal mucosa, and the incision can extend to the upper region of the nasal septum.^8^

### Olfaction assessment

The test chosen to carry out this study was based on the CCCRC test, which is carried out divided into two stages, as follows:

1. The threshold test: The threshold test utilizes successive dilutions of 1-butanol. The concentrations are: 4%, 1.3%, 0.44%, 0.15%, 0.049%, 0.016%, 0.0055%, 0.0018% and 0.00061% in deionized, nanopure water. The solutions are placed in 250 mL polyethylene bottles, containing 60 mL of the solution. As test control, same size bottles, containing water, are used. The two bottles are presented to the patient simultaneously, one containing the diluted butanol and the other containing the water, both without identification. The patients are asked to cover one of their nostrils with their own hand. The bottle is opened, and the patients are then asked to smell one container and then the other and indicate which of the bottles they perceive as having the most intense smell. If the answer is incorrect, one a higher concentration of butanol is promptly used. Four correct responses, with the same concentration, are necessary for the threshold to be identified. Then, the test is repeated in the contralateral nostril.^9^
2. Identification Test: It is carried out using a kit consisting of seven opaque 180 mL bottles containing talc powder, powdered chocolate, cinnamon sticks, powdered coffee, mothballs, crushed peanut candy and a soap bar. The patient is again asked to cover one nostril with his hand while the other is tested. The patients smell the contents of the bottle while they are given some options of what that smell might be, and they must choose one of the options.

In case of error, the patient will have a second chance at the end of the test in that same nostril. A correct response at this second chance overrides the first wrong answer. Responses such as: “I cannot smell anything” or “I don’t know” are accepted, which will be considered as wrong responses. The test is then performed in the second nostril.^9^

At the end of the test, the average threshold values for the detection of butanol obtained in each nasal cavity were calculated. The mean of the corrected values was also obtained from the qualitative test of the right and the left nasal cavities. Finally, an average is reached between the final value obtained in the qualitative and the quantitative test, which is the final value of the test.

This value will later be classified into one of the following categories: 0–1.75 – anosmia; 2–3.75 – severe hyposmia; 4–4.75 – moderate hyposmia; 5–5.75 – mild hyposmia; >6 – normosmia.^9^

### Statistical analysis

The Connecticut score classifications were established according to groups and in the total of patients using absolute and relative frequencies.^10^ The classifications between groups and evaluation moments were compared using Generalized Estimation Equations (GEEs), with an autoregressive correlation matrix of order 1 between the moments, with marginal Poisson distribution and identity link function. The analysis was followed by multiple Bonferroni comparisons to define between which groups or moments there are differences in the Connecticut score categories.

The analyses were performed using the software IBM-SPSS for Windows, version 20.0 and tabulated using the Microsoft-Excel 2003 software and the tests were performed with a 5% significance level.

## Results

The smell tests were applied to a total of 22 patients at the following moments: preoperative, 1 month, 3 months and 6 months post-operatively. However, one patient was excluded because the transcribriform access was performed, due to the diagnostic hypothesis of olfactory groove meningioma. Hence, 21 patients completed the study. None of the patients were lost to follow-up.

Subsequently, the patients were divided into two groups. Group 1 comprised a total of 17 patients (10 with non-functioning pituitary adenomas and 7 with functioning adenomas), who were submitted to transsphenoidal hypophysectomy. Group 2 included a total of 4 patients (2 meningiomas of the tuberculum sellae, 1 meningoencephalocele of the lateral sphenoid recess on the right and 1 craniopharyngioma). This division was made to elucidate a possible difference in the behavior of the two groups over the analyzed time. Considering that the pattern of results was similar in both groups over the analyzed period, it was possible, based on this observation, to group all patients into a single group to perform the statistical analysis.

The distribution in relation to gender and age in each of the groups is shown in Table 1, Table 2. Table 3, Table 4 depict the distribution of the results of the CCCRC smell test, according to the described groups.

**Table 1:** Patient distribution according to gender in Groups 1 and 2.

	Group 1	Group 2
Male	76.5% (13)	0
Female	23.5% (4)	100% (4)

**Table 2:** Age distribution of patients in Groups 1 and 2.

	Minimum age	Maximum age	Mean
Group 1	10	53	50.35
Group 2	38	51	44.4

**Table 3:** Distribution of patients in Group 1 in relation to the results obtained at the CCCRC smell test.

	Normosmia	Mild hyposmia	Moderate hyposmia	Severe hyposmia	Anosmia
Preoperative	13	1	0	3	0
1 month	2	1	1	9	4
3 months	13	1	0	2	1
6 months	14	0	1	2	0

**Table 4:** Distribution of patients in Group 2 in relation to the results obtained at the CCCRC smell test.

	Normosmia	Mild hyposmia	Moderate hyposmia	Severe hyposmia	Anosmia
Preoperative	4	0	0	0	0
1 month	1	0	1	0	2
3 months	3	0	0	1	0
6 months	3	0	0	1	0

**Figure 1:**
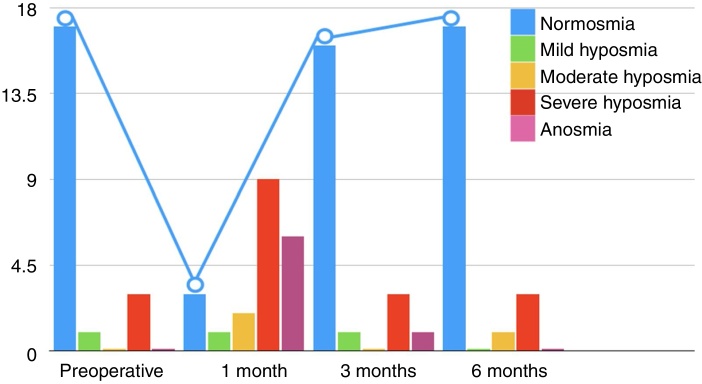
Description of the Connecticut score classifications in all patients over the 6-month follow-up period, showing how the smell worsens in the first postoperative month.

Fig. 1 suggests that, in the first month of patient follow-up, the sense of smell worsened, but that, during the follow-up, there was an improvement in the classification.

Table 5 shows that the average behavior of the groups during follow-up was statistically equal (*p* = 0.750) and there was no difference in the mean classifications between the groups (*p* = 0.726); however, between the evaluation moments, there was a mean difference in the classifications, regardless of the group (*p* < 0.001).

**Table 5:** Description of the Connecticut score classifications according to groups during the follow-up and result of the comparisons.

Connecticut	Group								P Group	P Moment	P Interaction
	Pituitary (*n* = 17)				Non-pituitary (*n*+)						
	Pre-op.	1 mo.	3 mo.	6 mo.	Pre-op.	1 mo.	3 mo.	6 mo.			
Anosmia	0 (0)	4 (23.5)	1 (5.9)	0 (0)	0 (0)	2 (50)	0 (0)	0 (0)	07.26	<0.001	0.750
Severe hyposmia	3 (17.6)	9 (52.9)	2 (11.8)	2 (11.8)	0 (0)	0 (0)	1 (25)	1 (25)			
Moderate hyposmia	0 (0)	1 (5.9)	0 (0)	1 (5.9)	0 (0)	1 (25)	0 (0)	0 (0)			
Mild hyposmia	1 (5.9)	1 (5.9)	1 (5.9)	0 (0)	0 (0)	0 (0)	0 (0)	0 (0)			
Normosmia	13 (76.5)	2 (11.8)	13 (76.5)	14 (82.4)	4 (100)	1 (25)	3 (75)	3 (75)			

EEG with Poisson distribution and identity link function.

Table 6 shows that the patients’ mean classification was statistically worse than in the other evaluation moments only in the first month of follow-up (*p* < 0.001). However, there was no mean difference in the Connecticut score classification between the other moments (*p* > 0.05); that is, patients worsened in the first month and returned to the preoperative classification on average in the third month of follow-up.

**Table 6:** Result of comparisons of the Connecticut score classifications between the moments of evaluation.

Comparison	Mean difference	Standard Error	gL	*p*	95% CI	
					Lower	Upper
Preoperative – 1 month	2.27	0.40	1	<0.001	1.21	3.33
Preoperative – 3 months	0.32	0.54	1	<0.999	−1.12	1.75
Preoperative – 6 months	0.23	0.59	1	<0.999	−1.33	1.79
1 month – 3 months	−1.95	0.39	1	<0.001	−2.98	−0.93
1 month – 6 months	−2.05	0.48	1	<0.001	−3.30	−0.79
3 months – 6 months	−0.09	0.44	1	<0.999	−1.24	1.06

Bonferroni multiple comparisons.

## Discussion

The sense of smell is very important for humans and, as the endoscopic endonasal approach to the skull base requires the resection of areas where there is olfactory neuroepithelium or regions close to it, it is important to consider whether this surgical approach will cause any damage, permanent or transient, to the patients’ sense of smell, since the loss of this sense, or at least its decrease, can cause important emotional, social and safety problems.^11^, ^12^

During the follow-up of the patients in the present study, we observed that, in the return consultations for the evaluation of the nasal cavity, they showed, especially during the 1^st^ month after surgery, an important inflammatory process and the presence of nasal crusts, due to extensive surgical manipulation. Over the months, this process showed a favorable evolution, through cleaning performed during consultations and nasal washing with 0.9% saline on free demand, resulting in improved olfaction and nasal air flow. These factors may explain the worsening of olfaction observed in the first month after surgery in relation to preoperative rates, followed by normalization three months after the surgery.

These findings corroborate the study by Hart et al., who described the same changes in the nasal cavity in the first month after the surgery, which alter the nasal flow and block the olfactory neuroepithelium, causing hyposmia or conductive anosmia. The worsening of the patients’ sense of smell in the postoperative period of endonasal skull base surgery is, therefore, related to direct trauma to the olfactory epithelium, since the inflammatory process alters both the mucosa structure and the mucus composition, as well as obstructive problems that prevent the air flow from reaching this epithelium.^11^

Data on transient olfactory dysfunction after endoscopic skull base surgery are still conflicting in the literature^4^, ^5^, ^8^, ^12^, ^13^, ^14^, ^15^, ^16^, ^17^, ^18^; however, Yin et al. conducted a meta-analysis with 29 articles in 2019 and demonstrated that the objective tests showed no statistically significant change between the preoperative and long-term postoperative olfactory function. They concluded there is a transient change in olfaction in the first postoperative month, which normalizes between the 3^rd^ and 6^th^ months after the surgery. However, the same authors were unable to collect enough data to carry out a meta-analysis comparing patients in whom the nasoseptal flap was used or not.^19^

Another controversial information in the literature is related to the removal of the middle turbinate, as this structure has olfactory neuroepithelium. Therefore, many authors warn that the removal of this structure during access to the skull base can alter the nasal function, reducing the sense of smell.^20^, ^21^, ^22^

The present study carried out a careful inclusion selection, which made the series homogeneous regarding tumor access (nasoseptal and reverse flap, partial removal of the middle and upper turbinate, unilateral anterior and posterior ethmoidectomy), concluding that, after three months, the olfactory function returns to preoperative values. The same result was demonstrated in the studies by Sowerby et al.^4^ and Upadhyay et al.,^8^ in which the olfactory function was preserved by performing the same surgical technique of partial removal of the middle turbinate. Sowerby et al. also performed partial removal of the upper turbinate, as in the present study.

This result allows us to affirm that with the preservation of some areas of the olfactory neuroepithelium or without complete damage to the latter, smell does not suffer permanent harm. When the basilar layer of the mucosa is completely damaged, metaplastic substitution occurs, with epithelium similar to the respiratory one; on the other hand, when the damage is not significant, it regenerates, with a smaller number of cells, which become irregularly arranged.^23^

Aiming to avoid olfactory loss, some authors have offered a modification of the original technique for creating the nasoseptal flap. Harvey et al. proposed, in the making of the nasoseptal flap, performing the upper incision at a lower level in the nasal septum region, sparing 50% of the mucosa in the upper region, compared to the original technique.^14^ Another suggestion is the use of a cold steel scalpel to make the upper incision in the nasal septum region, which would help to reduce epithelial damage and decrease the damage to the olfactory region, since monopolar cautery can disseminate energy during the incision and damage adjacent regions.^24^

The results obtained in the present study are the same as those found in the study by Harvey et al., corroborating the idea that the original technique is capable of preserving the olfactory epithelium present in the upper region of the nasal septum.^14^ It is important to point out that, in cases of more extensive defects of the anterior skull base, the reduction of the nasoseptal flap may be insufficient to avoid frequent causes of morbidity of this access route, such as CSF fistula and meningitis.

In this study, we recommend using the reverse flap, which, when covering the bare cartilaginous septum (ipsilateral to the nasoseptal flap), provides an earlier re-epithelialization, in approximately two weeks, and less formation of nasal crusts. Jo et al. described that healing by second intention, without the reverse flap, takes up to three months for full recovery.^25^

In the present study, although patients with heterogeneous pathologies were included, among them pituitary adenomas, meningiomas, craniopharyngiomas and lateral recess fistula of the sphenoid sinus, the surgical technique employed, the access route, and the postoperative follow-up were homogeneous. This standardization reduces the occurrence of bias and facilitates the comparison with future studies. The literature is still scarce regarding studies on the olfaction of patients submitted to medium turbinectomy and, concomitantly, use of the nasoseptal flap.

There is a great divergence in the literature regarding the interventions carried out by each group of researchers. The technique used in our service combines measures to better expose the sellar region (turbinectomies) with others to contain damage (flaps), but with care for maximum preservation of olfactory areas. In theory, greater interventions could result in significant olfaction impairment in relation to studies with a more conservative surgical technique. However, our results were similar.

Initially, we divided patients into two groups, those who underwent transsphenoidal surgery and those who required an expanded approach to the skull base, as there could theoretically be differences in the pattern of postoperative olfactory function due to different extensions of surgical access. However, as shown in Table 5, no such difference was observed, and all patients were joined in a single group and we concluded that, with the technique used by our group, there was no association between the access extent and the degree or duration of olfactory loss, normalized three months after the surgery. Considered together, these findings indicate the probable importance of the obstructive and inflammatory components that result in the temporary pattern of olfactory dysfunction found in the postoperative period of anterior skull base surgeries.^11^

The main study limitations are related to the small number of participants and the absence of a control group for the main interventions performed, such as turbinectomies and the creation of nasoseptal and reverse flaps, thus the impact of each cannot be specifically measured. Since anterior skull base surgery is an area undergoing remarkable expansion, further studies should be encouraged to better understand the measures necessary to preserve olfactory function.

## Conclusion

The endoscopic skull base surgery is constantly evolving and expanding its boundaries. As a result, more potential damage and anatomical changes can occur in the nasal fossa, thus leading to greater nasal morbidity, which includes olfactory dysfunction. In this study, we showed that the decrease in olfaction is a transient condition, normalizing in the third month in patients undergoing surgery, which technique includes creating the nasoseptal and reverse flaps, partial removal of the middle and upper turbinates and unilateral anterior and posterior ethmoidectomy.

## Conflicts of interest

The authors declare no conflicts of interest.
